# An Improved Localization Method for the Transition between Autonomous Underwater Vehicle Homing and Docking

**DOI:** 10.3390/s21072468

**Published:** 2021-04-02

**Authors:** Ri Lin, Feng Zhang, Dejun Li, Mingwei Lin, Gengli Zhou, Canjun Yang

**Affiliations:** 1 State Key Laboratory of Fluid Power and Mechatronic Systems, Zhejiang University, Hangzhou 310027, China; 11925004@zju.edu.cn (R.L.); li_dejun@zju.edu.cn (D.L.); lmw@zju.edu.cn (M.L.); zhougengli163@163.com (G.Z.); ycj@zju.edu.cn (C.Y.); 2 Advanced Technology Institute, Zhejiang University, Hangzhou 310027, China; 3 Pilot National Laboratory for Marine Science and Technology, Qingdao 266000, China

**Keywords:** autonomous underwater vehicle, AUV docking, ORBSLAM, underwater visual odometry, localization based on multi-sensor information fusion

## Abstract

Docking technology for autonomous underwater vehicles (AUVs) involves energy supply, data exchange and navigation, and plays an important role to extend the endurance of the AUVs. The navigation method used in the transition between AUV homing and docking influences subsequent tasks. How to improve the accuracy of the navigation in this stage is important. However, when using ultra-short baseline (USBL), outliers and slow localization updating rates could possibly cause localization errors. Optical navigation methods using underwater lights and cameras are easily affected by the ambient light. All these may reduce the rate of successful docking. In this paper, research on an improved localization method based on multi-sensor information fusion is carried out. To improve the localization performance of AUVs under motion mutation and light variation conditions, an improved underwater simultaneous localization and mapping algorithm based on ORB features (IU-ORBSALM) is proposed. A nonlinear optimization method is proposed to optimize the scale of monocular visual odometry in IU-ORBSLAM and the AUV pose. Localization tests and five docking missions are executed in a swimming pool. The localization results indicate that the localization accuracy and update rate are both improved. The 100% successful docking rate achieved verifies the feasibility of the proposed localization method.

## 1. Introduction

Due to its characteristics of high pressure, electromagnetic wave shielding and complex topography, it is a great challenge to explore and develop the ocean. So far, scientists have put forward many observation methods. Among them, cabled ocean observation networks (COONs) and autonomous underwater vehicles (AUVs) are the most well-known of these observation methods. A COON supplies electric energy and exchanges data with various detection sensors placed on the seabed through a junction box [1]. The AUV which carries own energy system and various detection sensors can navigate autonomously to execute observation missions. In order to combine the above two observation methods, underwater docking technology is proposed which effectively extends the observation time and range of the AUVs [2,3,4,5]. One of the key technologies is AUV navigation. Generally, navigation and localization systems on land mainly include GPS-based navigation, lidar navigation, electromagnetic navigation and visual navigation. Visual navigation has the advantages of abundant and accurate information, small cooperative interference and strong real-time performance. Simultaneous localization and mapping (SLAM) is widely used in visual navigation system, which can not only estimate their own pose, but also create environment maps. Since the development of underwater vehicles, the navigation methods can be roughly divided into three categories [6]: geophysical navigation methods, dead reckoning or multi-data coupling navigation methods and navigation methods based on acoustic sensors. Marine geophysical navigation stores the spatial distribution of the geomagnetic field, gravity field, optical signals or depth and other parameters near the navigation area into the main control unit of the AUV in advance, so that the AUV can detect these physical quantities in real time during navigation to match with the data of storage unit, so as to correct the navigation position of the AUV. For AUVs, the most commonly used navigation method is dead reckoning or multi-data coupling navigation [7,8]. Both dead reckoning and coupled navigation methods will encounter the problem of cumulative error, also known as time drift. Acoustic positioning sensors can provide absolute position information to AUVs underwater, and help AUVs correct their position [9]. Common acoustic localization sensors include long baseline, short baseline and ultra-short baseline (USBL). According to the distance between AUV and docking station, the returning process of AUVs is generally divided into two stages: remote homing (more than 15 m from the docking station) and terminal docking (10~20 m from the docking station) [10,11]. When the AUV is in the remote homing phase, usually it only needs to travel in the direction to get close to the docking station, and the navigation accuracy requirement is not very high. Generally, the acoustic navigation commonly uses an ultra-short baseline (USBL) localization system. In terminal docking, due to the short distance between the AUV and the docking station, more accurate navigation methods with good real-time performance are required. Acoustic navigation using USBL, optical navigation using a camera and a light and electromagnetic navigation are usually utilized in this stage [3,6,12]. Although the navigation methods mentioned above all use two or more sensors, in fact, in different navigation stages, often one kind navigation sensor is used, that is, an acoustic sensor is used in the remote homing, and an optical sensor is used in the terminal docking phase [13,14,15]. We can find that either an acoustic sensor or a visual sensor is applied in terminal docking. When the acoustic sensor such as USBL is adopted in terminal docking, the low updating rate and outliers influence the AUV localization accuracy. In fact, between AUV homing and docking, there is a transition, where the localization performance also greatly affects the rate of successful docking. Due to the low localization rate and outliers of USBL navigation, the AUV may not see the optical navigation guidance light of after entering the transition zone and the distance between the AUV and docking station where the camera can distinguish the guidance light is affected by the ambient light variation. By the time the AUV distinguishes the guidance light, the distance may be too short and the AUV does not have enough time to adjust its pose for better docking. Even worse, the camera could not see the light at all. In this situation, the localization can only rely the last data of the USBL, which possibly results in docking failure.

Therefore this paper applies ORBSLAM-related technology and proposes an improved localization method based on multi-sensor information fusion of a visual odometer, USBL, attitude and heading reference system (AHRS) and a depth meter in the transition between AUV homing and docking. ORBSLAM is the epitome of SLAM technology of feature point methods, which have excellent performance on land [16,17]. The ORBSLAM algorithm is developed from the parallel tracking and mapping (PTAM) algorithm [18,19,20,21]. It is one of the relatively perfect and easy to use algorithms among SLAM algorithms. ORB feature points are used as feature points in ORBSLAM, and the real-time feature extraction and accuracy of feature matching are considered. Moreover, ORBSLAM has many improvements in engineering implementation, such as ensuring the uniform distribution of extracted features, and getting more accurate results by multiple optimization when optimizing poses, so it has strong adaptability to complex scenes. Monocular ORBSLAM algorithms are divided into three threads: tracking, local mapping and loop detection. However, when a AUV is working underwater, it will inevitably encounter sudden changes in light and ocean currents, resulting in the occurrence of feature point mismatching. To improve the underwater working ability of the ORBSLAM, an improved underwater ORBSLAM (IU-ORBSLAM) for the transition between AUV homing and docking is proposed, which has better continuous working ability in the situation of light changes and motion mutations. Then, a nonlinear optimization based multi-sensor information fusion localization method is proposed, which includes the information of monocular visual odometry in IU-ORBSLAM, USBL, AHRS and a depth meter. It solves the scale issue of the monocular odometer and improves the localization accuracy and update rate during the transition.

The rest of the paper is described as follows: Section 2 describes the proposed IU-ORBSLAM in the transition between AUV homing and docking. The bags of words, RANSAC algorithm and redundant multilayer tracking framework are used in the proposed IU-ORBSLAM to improve the localization performance of AUV monocular vision. Section 3 describes the improved localization methods based on multi-sensor information fusion of monocular visual odometry, USBL, depth meter and attitude and AHRS. The definition of navigation coordinates and nonlinear optimization method are introduced. The sum of residuals of each sensor is taken as the cost function to optimize the AUV’s pose and the scale coefficient of monocular visual odometry. Section 4 demonstrates the experimental results and Section 5 draws conclusions and discusses possible future work.

## 2. IU-ORBSLAM for the Transition between AUV Homing and Docking

### 2.1. Problems in ORBSLAM

This paper mainly studies the localization of AUV based on fusion of the visual odometry, depth meters, AHRS and USBL in the transition between AUV homing and docking. In this situation, the information fusion algorithm can correct the AUV’s pose errors. The AUV docking route is generally unidirectional, so the loop detection and correction is not significant. The visual odometry of ORBSLAM in the tracking thread is mainly studied here. The pose of the AUV may suddenly change if a current change or light variation occur when using ORBSLAM underwater. The motion velocity of the present frame can be quite different from that of the last frame. When the motion model is used to track the last frame, the projection of the feature points of the last frame in the present frame are far away from the real matching points. This means that the number of feature matches is small and there are mismatches.

**Figure 1:**
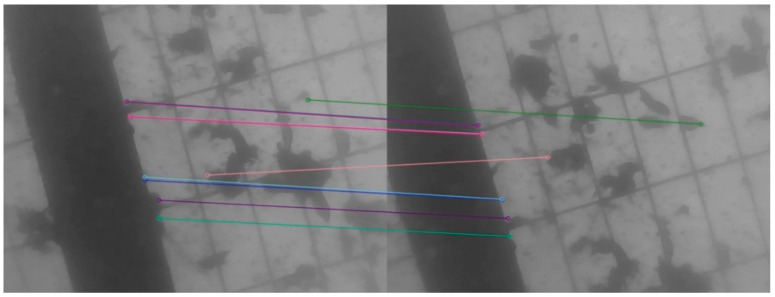
The matching point pairs between the present and the last frame by using the motion model. There are few matching point pairs and some matching point pairs are false.

As shown in Figure 1, due to the small number of matching pairs, the number of matching points on the map is below the threshold. According to the tracking logic of ORBSLAM, tracking the reference key frame is executed next. However, due to motion changes, the number of feature points that match between the last frame and the reference key frame is also small. The number of matching point pairs is lower than the threshold, which results in tracking failure. Even if the threshold is reduced so that the tracking key frame can be implemented, there will be many mismatches in the feature point matching, resulting in a larger camera pose estimation error and a larger map point error. Thus, with the tracking going on, the number of tracking features will be smaller, and the tracking error will be larger, and finally the tracking fails.

In order to solve these two problems in ORBSLAM, this paper proposes IU-ORBSLAM. In IU-ORBSLAM, the method of tracking the last frame based on bags of words is adopted. Then mismatching elimination is added and finally, a multi-level redundant tracking framework is proposed.

### 2.2. Tracking the One Last Frame Based on Bags of Words

**Figure 2:**
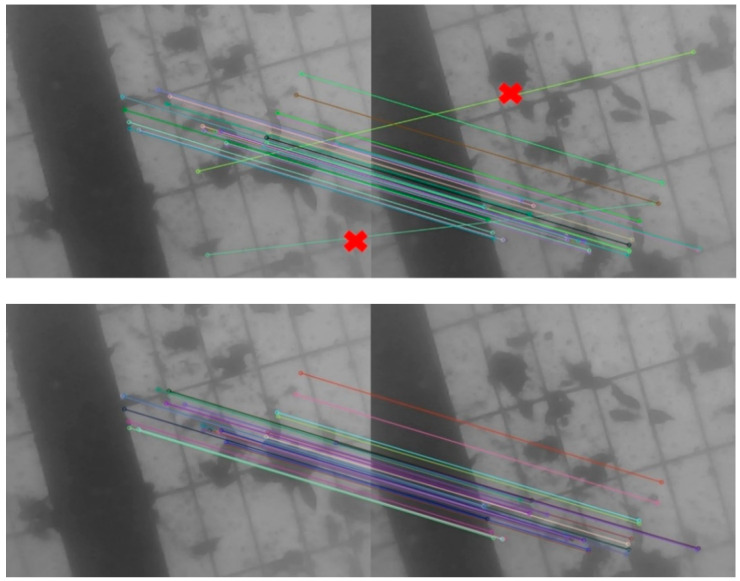
The matching point pairs between the present and the last frame after using bags of words and RANSAC. The up one shows the matching pairs only using bags of words. The pairs are greatly increased. However, there are two mismatches marked by red×. The bottom one shows the matching point pairs after eliminating mismatches in the up one figure.

The DBOW2 algorithm is an excellent open source project because it can build vocabulary trees, calculate image similarity and detect loops [16]. DBOW2 clusters feature descriptors into multiple words based on a K-means + + clustering algorithm [21], and constructs a word tree according to the data structure of the tree. The vocabulary tree is based on a K-means + + algorithm, so it can carry out fast feature matching. This feature matching method is called bags of words matching in this paper. Based on matching by bags of words, feature points are extracted from the last frame. The descriptors are calculated to obtain the image feature points set. Then, the DBOW2 algorithm is used to find and classify each feature point. Similarly, the present frame is also classified. In feature matching of two images, the classified feature categories are used for comparison. Then the Hamming distance of the calculated descriptors is matched with the features of the same category. Feature category is essentially a number, while the descriptor is a vector. The matching speed of one-dimensional number is much faster than that of high-dimensional vector, so it can greatly improve the matching speed. Since the matching range is not constrained by the motion relationship, more feature points can be matched in the image, as shown in Figure 2. Compared with Figure 1, more correct matches can be obtained by tracking the last frame based on bags of words. However, there are still a few mismatching, as shown in the red mark in Figure 2, which needs to be further eliminated by a random sample consensus (RANSAC) algorithm [22,23] (Figure 2).

### 2.3. Redundant Multilayer Tracking Framework

**Figure 3:**
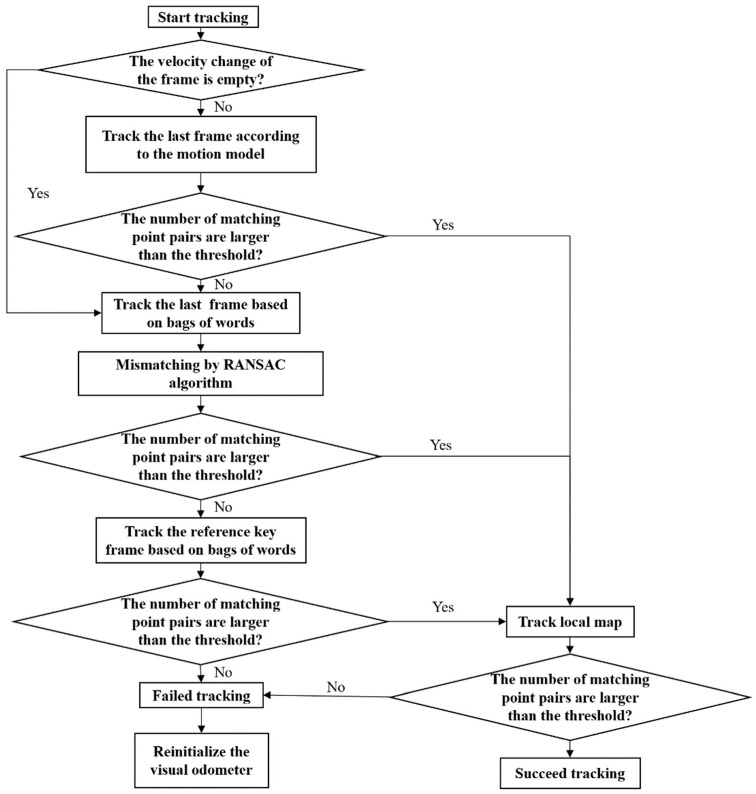
Redundant multilayer tracking framework in IU-ORBSLAM.

Based on the ORBSLAM tracking framework, IU-ORBSLAM adds bags of words to track the last frame and RANSAC to eliminate mismatches. The tracking logic is redesigned to implement a redundant multi-layer tracking framework (Figure 3). Under normal circumstances, the motion model is used to track the last frame. When a motion change or light variation occurs, the number of matching features in the last frame is less than the threshold value. Then the tracking of last frame based on bags of words will be executed. At the same time, the mismatches are eliminated by RANSAC to ensure that the last frame can be tracked correctly, which greatly avoids missing matching points. The framework retains the reference key frame and local map of ORBSLAM, which enhances the redundancy of the tracking framework and the ability of tracking feature points. In addition, there is no response after ORBSLAM fails to track, but the IU-ORBSLAM will initialize and restart. Under this tracking framework, the continuous motion ability of IU-ORBSLAM in underwater environment can be improved.

## 3. Multi-Sensor Information Fusion Localization Method Based on Nonlinear Optimization

Scale estimation is one of the key problems in the applications of monocular visual odometry. Due to the lack of scale information in monocular vision, the relative pose changes can only be obtained from two adjacent frames, which leads to cumulative error (or cumulative drift). In this paper, a nonlinear optimization method is proposed for information fusion of underwater monocular visual odometry, USBL, depth meter data and AHRS. The pose of the AUV and the scale coefficient of monocular visual odometry are simultaneously taken as variables to be optimized. The data of USBL is applied to provide scale information for monocular visual odometry which suppresses the cumulative drift. The monocular visual odometry is used to improve the update rate of localization data and smooth the abnormal value of the USBL. Since the absolute position, depth and attitude of AUV are obtained by the USBL, depth meter and AHRS, respectively, there is no cumulative error but rather just the absolute error of each sensor. After fusion, not only is the monocular scale determined, but also the pose estimation accuracy and data updating rate are improved. In order to evaluate the localization accuracy, the UWB localization module with high localization accuracy (the localization error is less than 10 cm after calibration) is selected to measure the real navigation trajectory of AUV, which is taken as the reference trajectory. The model of the UWB module used in this paper is D-DWM-PG1.7. The coverage area of two-dimensional localization can reach 35 m × 35 m. And the localization accuracy in this covered area is 10 cm. There are three UWB localization stations, one of which is installed on a right angle point of the swimming pool, and the other two are installed on two sides of the right angle, respectively, and they are 21.8 m and 12 m away from the UWB base station on the right angle point, respectively. The UWB sensor does not conduct multi-sensor data fusion.

### 3.1. Definition of Navigation Coordinate

**Figure 4:**
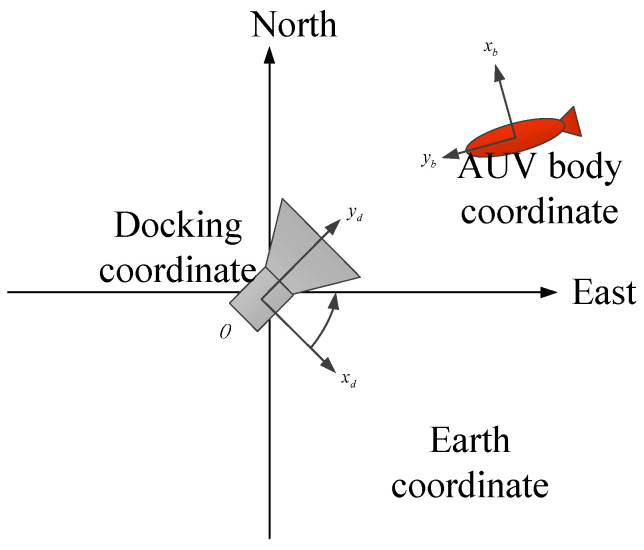
Definition of AUV body coordinate, docking coordinate and geodetic coordinate.

A localization method of multi-sensor information fusion for the transition mainly uses a monocular camera, USBL, AHRS and a depth meter. As shown in Figure 4, the docking coordinate is a right-hand coordinate established by taking the center of the docking station as the origin. The docking coordinate is taken as the reference coordinate for navigation. The localization information of AUV in the docking coordinate is obtained by USBL. The geodetic coordinate usually is the East-North-Up (ENU) coordinate. In order to conveniently select the origin of the geodetic coordinate at the center of the docking station, the longitude and latitude obtained by GPS can usually be converted into the localization information under the geodetic coordinate. The AUV body coordinate is established with the floating center of AUV as the origin. The coordinate of the camera and AHRS carried by AUV are shown in Figure 4.

**Figure 5:**
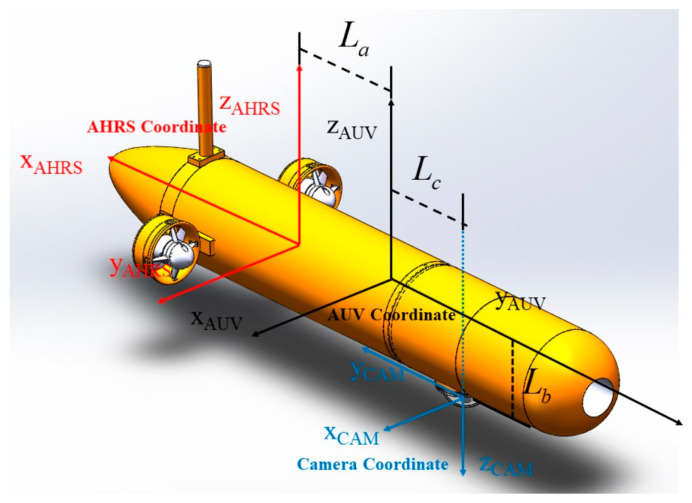
Relationship among AUV body coordinate, AHRS coordinate and camera coordinate.

According to the coordinate defined in Figure 5, the transformation matrix from the AUV body coordinate to the camera coordinate is:

(1) $$T_{AUV\_CAM}=\left[\begin{array}{cccc} 1 & 0 & 0 & 0\\ 0 & -1 & 0 & L_{c}\\ 0 & 0 & -1 & -L_{b}\\ 0 & 0 & 0 & 1 \end{array}\right],$$

and the transformation matrix from the AHRS coordinate to the AUV body coordinate is:

(2) $$T_{AHRS\_AUV}=\left[\begin{array}{cccc} 0 & -1 & 0 & 0\\ 1 & 0 & 0 & 0\\ 0 & 0 & 1 & 0\\ 0 & 0 & 0 & 1 \end{array}\right],$$

The Euler angle measured by the AHRS is based on the West-South-Up (WSU) coordinate, and the rotation order of Euler angle is z-y-x, corresponding to heading angle, pitch angle and roll angle. Defining the heading angle, pitch angle and roll angle of the AHRS are ϕ,θ,ψ respectively, the corresponding rotation matrix is $R_{WSU\_AHRS}$, which is expressed as follows:

(3) $$\left[\begin{array}{ccc} \cos\theta \cos\phi & \sin\psi \sin\theta \cos\phi -\cos\psi \sin\phi & \cos\psi \sin\theta \cos\phi +\sin\psi \sin\phi \\ \cos\theta \sin\phi & \sin\psi \sin\theta \sin\phi +\cos\psi \cos\phi & \cos\psi \sin\theta \sin\phi -\sin\psi \cos\phi \\ -\sin\theta & \sin\psi \cos\theta & \cos\psi \cos\theta \end{array}\right],$$

The corresponding homogeneous transformation matrix is $T_{WSU\_AHRS}$:

(4) $$\left[\begin{array}{cccc} \cos\theta \cos\phi & \sin\psi \sin\theta \cos\phi -\cos\psi \sin\phi & \cos\psi \sin\theta \cos\phi +\sin\psi \sin\phi & 0\\ \cos\theta \sin\phi & \sin\psi \sin\theta \sin\phi +\cos\psi \cos\phi & \cos\psi \sin\theta \sin\phi -\sin\psi \cos\phi & 0\\ -\sin\theta & \sin\psi \cos\theta & \cos\psi \cos\theta & 0\\ 0 & 0 & 0 & 1 \end{array}\right],$$

After rotating an anticlockwise degree of $\phi_{DOCK\_WSU}$ around the Z axis, the docking coordinate coincides with the WSU coordinate. Therefore, the transformation matrix from the docking coordinate to the WSU coordinate is $T_{DOCK\_WSU}$:

(5) $$\left[\begin{array}{cccc} \cos\phi_{DOCK\_WSU} & -\sin\phi_{DOCK\_WSU} & 0 & 0\\ \sin\phi_{DOCK\_WSU} & \cos\phi_{DOCK\_WSU} & 0 & 0\\ 0 & 0 & 1 & 0\\ 0 & 0 & 0 & 1 \end{array}\right],$$

According to the coordinate transformation relationship, the attitude of AUV in docking coordinate can be obtained through AHRS, that is, the rotation matrix $R_{DOCK\_AUV}$ from the docking coordinate to the AUV body coordinate:

(6) $$R_{DOCK\_AUV}={\left[T_{DOCK\_AUV}\right]}_{1:3,1:3}={\left[T_{DOCK\_WSU}\cdot T_{WSU\_AHRS}\cdot T_{AHRS\_AUV}\right]}_{1:3,1:3},$$

The longitude and latitude of GPS are converted into the position of AUV in geodetic coordinate by using the following empirical formula:

(7) $$\begin{array}{l} R_{a}=6378137\\ R_{b}=6356752\\ dR=\frac{R_{b}\sqrt{1+\tan^{2}(lat)}}{\sqrt{{\left(R_{b}/R_{a}\right)}^{2}+\tan^{2}(lat)}}\\ gps\_x=dR\cdot \sin(lon-lon\_dock)\cos(lat)\\ gps\_y=dR\cdot \sin(lat-lat\_dock) \end{array},$$

where lon_dock,lat_dock are the longitude and latitude of the docking station, lon,lat are the longitude and latitude of the AUV, respectively. (gps_x,gps_y) denotes the position of AUV in geodetic coordinate. After rotating an anticlockwise degree of $\phi_{DOCK\_WORLD}$ around the Z axis, the docking coordinate coincides with the geodetic coordinate. Therefore, the transformation matrix from the docking coordinate to the geodetic coordinate is:

(8) $$T_{DOCK\_WORLD}=\left[\begin{array}{cccc} \cos\phi_{DOCK\_WORLD} & -\sin\phi_{DOCK\_WORLD} & 0 & 0\\ \sin\phi_{DOCK\_WORLD} & \cos\phi_{DOCK\_WORLD} & 0 & 0\\ 0 & 0 & 1 & 0\\ 0 & 0 & 0 & 1 \end{array}\right],$$

According to the coordinate transformation relationship in Formulas (5) and (8), the position of AUV in the docking coordinate can be obtained by GPS and depth meter:

(9) $$\begin{array}{ll} t_{DOCK\_AUV} & ={\left[T_{DOCK\_WORLD}\cdot t_{WORLD}\right]}_{1:3,1}\\ & =\;{\left[T_{DOCK\_WORLD}\cdot {\left[\begin{array}{cccc} gps\_x & gps\_y & depth\_dock-depth\_auv & 1 \end{array}\right]}^{T}\right]}_{1:3,1} \end{array},$$

where depth_auv and depth_dock are the depth of AUV and docking station, respectively. For USBL, the position matrix $t_{DOCK\_AUV}$ of AUV in the docking coordinate can be obtained directly. Due to the limitation of test conditions, the position of AUV in the docking coordinate obtained by GPS and depth meter instead of USBL. By using Equations (6) and (9), the position and attitude of AUV in the docking coordinate calculated by the combination of AHRS, GPS and depth meter are obtained:

(10) $$T_{DOCK\_AUV}=\left[\begin{array}{cc} R_{DOCK\_AUV} & t_{DOCK\_AUV}\\ 0 & 1 \end{array}\right],$$

### 3.2. State Estimation Based on Nonlinear Optimization

In the vision based multi-sensor information fusion, the state set to be estimated is assumed to be $X_{n}={\left\{x_{i}\right\}}_{i=1,2,\cdots n}$. The observation set is $Z_{m}={\left\{z_{j}\right\}}_{j\in 1,2,\cdots ,m}$. The state subscripts related to $z_{j}$ constitute the set $S_{j}$. The states related to $z_{j}$ constitute the set $A_{j}={\left\{x_{k}\right\}}_{k\in S_{j}}$. Since an observation is related to several state variables, the observation equation is:

(11) $$z_{j}=f_{j}(A_{j})+v_{j},$$

where $f_{j}$ is the observation function. Assuming that the observation noise obeys Gaussian distribution $v_{j}\sim Ν(0,\sum_{j})$, where $\sum_{j}$ is the noise covariance, then:

(12) $$P(z_{j}|A_{j})=P((z_{j}-f_{j}(A_{j}))|A_{j})=\exp(-\frac{1}{2}||(z_{j}-f_{j}(A_{j}))|{|}_{\sum_{j}}^{2}),$$

According to Equation (12) and assuming that each observation is independent of each other, $X_{0}$ is used to represent the prior of $X_{n}$. And the prior distribution is also assumed to obey Gaussian distribution $X_{0}$, then:

(13) $$\begin{array}{ll} X_{n}^{*} & =\underset{X_{n}}{\arg\max}P(X_{n}|Z_{m})\\ & =\underset{X_{n}}{\arg\min}\left(||e_{0}{|}_{\sum_{0}}^{2}+\sum_{j}||e_{j}|{|}_{\sum_{j}}^{2}\right) \end{array},$$

where $e_{0}\sim X_{0}-\mu_{0}$ and $e_{j}\sim z_{j}-f_{j}(X_{j})$ are the prior residuals and the observed residuals, and $||e|{|}_{\sum }≐e^{T}\sum^{-1}e$ are the Mahalanobis distances with the variance of ∑. By Equation (13), the posterior probability maximization problem is transformed into a nonlinear least square optimization problem. The cost function can be integrated into:

(14) $$E(X)=||e_{0}{|}_{\sum_{0}}^{2}+\sum_{j}||e_{j}|{|}_{\sum_{j}}^{2}=||e(X)|{|}_{\sum }^{2},$$

where e(X) is the extended residual vector and ∑ is the extended covariance matrix, which satisfies the following conditions:

(15) $$\begin{array}{l} e(X)=\left[\begin{array}{c} e_{0}\\ e_{1}\\ \vdots \\ e_{m} \end{array}\right]=\left[\begin{array}{c} \mu_{0}-X_{0}\\ z_{1}-f_{1}(X_{1})\\ \vdots \\ z_{m}-f_{m}(X_{m}) \end{array}\right]\\ \sum^{-1}=\left[\begin{array}{cccc} \sum_{0}^{-1} & & & \\ & \sum_{1}^{-1} & & \\ & & \ddots & \\ & & & \sum_{m}^{-1} \end{array}\right] \end{array},$$

If Equation (13) is replaced by Equation (14), the least squares estimation is:

(16) $$X_{n}^{*}=\underset{X_{n}}{\mathrm{argmin}}(||e(X)|{|}_{\sum }^{2}),$$

The state estimation of multi-sensor information fusion can be obtained by solving the least square estimation. The key is to calculate the residuals of the sensors used.

### 3.3. Optimization Method

#### 3.3.1. The Pose Map Model of Multi Sensor Information Fusion Localization

According to the characteristics of the applied sensors, they can be divided into local localization sensors and global localization sensors. Local sensor, represented by monocular camera, mainly estimates the motion increment of AUV in local reference frame. Its main characteristic is that it has high relative motion measurement accuracy but large cumulative state error. And the monocular vision also lacks scale information. The global sensor, represented by USBL, depth meter and AHRS, mainly measures the absolute state of AUV in the global environment. Its main feature is that there is no cumulative error, but it has measurement noise and low update rate, especially USBL. The fusion of local localization sensors and global localization sensors can obtain accurate global pose [24]. Therefore, in this paper, monocular visual odometry, USBL, depth meter and AHRS are fused to achieve higher localization accuracy and higher update rate.

The camera attitude and scale-free displacement sequences obtained by monocular visual odometry are:

(17) $$\alpha =\left\{x_{0}^{VO},x_{1}^{VO},\cdots ,x_{l}^{VO}\right\},$$

(18) $$x_{i}^{VO}=\left\{p_{i}^{VO},q_{i}^{VO}\right\},$$

where $p_{i}^{VO},q_{i}^{VO}$ are the scale-free position of the camera corresponding to the *i*-th frame and the attitude expressed in quaternion form in the camera coordinate. The position and attitude sequences of AUV are obtained by the USBL, depth meter and AHRS, respectively,

(19) $$\begin{array}{l} \beta =\left\{p_{0}^{D},p_{1}^{D},\cdots ,p_{j}^{D}\right\}\\ \chi =\left\{q_{0}^{D},q_{1}^{D},\cdots ,q_{k}^{D}\right\} \end{array},$$

where $p_{j}^{D},q_{k}^{D}$ represent the position of the AUV at time j observed in the docking coordinate and the attitude of the AUV at time k in the docking coordinate. USBL can obtain the position of AUV relative to docking station. The depth meter can obtain the height of AUV relative to docking station.

In the multi-sensor information fusion-based monocular vision, one aim is to estimate the position and attitude of AUV relative to the docking coordinate system. Therefore, the position and attitude $x_{i}^{AUV}$ of AUV in the docking coordinate is selected as the estimated variable. Due to the lack of scale for the output position of monocular visual odometry, the scale coefficient s is also taken as the estimated variable. Let the set of variables to be optimized be $X=\left\{x_{0}^{AUV},x_{1}^{AUV},\cdots ,x_{l}^{AUV},s\right\}$, where $x_{i}^{AUV}=\left\{p_{i}^{AUV},q_{i}^{AUV}\right\}$ represents the position and attitude of the AUV in the docking coordinate at time i, and s represents the scale coefficient. According to Formula (16), the optimized function is:

(20) $$X^{*}=\underset{X}{\arg\min}(||e(X)|{|}_{\sum }^{2}),$$

According to the sensors used, the cost function can be expanded as follow:

(21) $$\begin{array}{ll} e(X) & =e^{VO}+e^{USBL}+e^{AHRS}+e^{Depth}\\ & =\sum_{j}\left(||e_{j}^{VO}|{|}_{\sum_{V}}^{2}+||e_{j}^{USBL}|{|}_{\sum_{USBL}}^{2}+||e_{j}^{AHRS}|{|}_{\sum_{AHRS}}^{2}+||e_{j}^{Depth}|{|}_{\sum_{Depth}}^{2}\right) \end{array},$$

where $e^{VO}$ is the monocular visual odometry observation residual, $e^{USBL}$ is the USBL observation residual, $e^{AHRS}$ is the AHRS observation residual, and $e^{Depth}$ is the depth meter observation residual. The key is to determine the residual error of each sensor, and then solve the least square problem to obtain the pose and scale coefficient of AUV.

**Figure 6:**
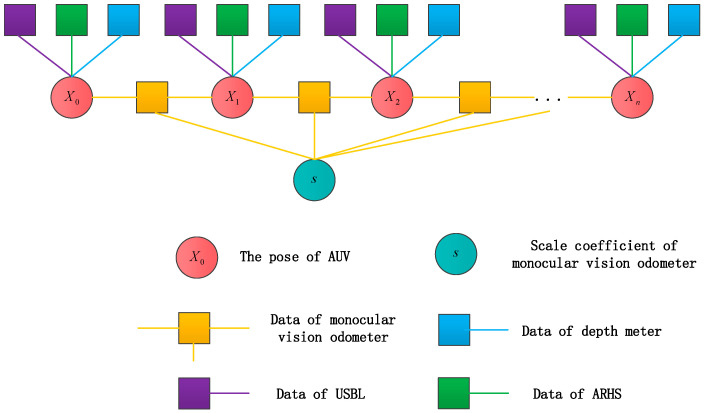
Optimized pose map.

In order to describe the relationship of multi-sensor information fusion more clearly, the pose map [23] is used for illustration, as shown in Figure 6. The circle nodes represent the variable to be optimized, where the red circle nodes represent the pose of AUV at each time, and the cyan circle nodes represent the scale coefficient of monocular visual odometry. The square nodes represent data of various sensors. The orange edges represent constraints in visual odometry. As a local sensor, it is an incremental constraint between the positions and orientations of AUV at adjacent times. The purple, green and blue edges represent constraints in USBL, AHRS and depth meter, respectively. These three sensors are absolute constraints on the position and attitude of AUV in the global coordinate system.

#### 3.3.2. Residuals of Sensors

The monocular visual odometry obtains the pose of the camera in the coordinate. However, the displacement measured is not the real displacement, but is proportional to the real displacement. Since the visual odometry is accurate in local area, so it mainly uses the pose between two adjacent frames to define the residual:

(22) $$\begin{array}{ll} e_{j}^{VO} & =z_{j}^{VO}-f_{j}^{VO}(X)\\ & =z_{j}^{VO}-f_{j}^{VO}(x_{j-1},x_{j})\\ & =\left[\begin{array}{c} sq_{j-1}^{VO}{}^{-1}(p_{j}^{VO}-p_{j-1}^{VO})\\ q_{j-1}^{VO}{}^{-1}q_{j}^{VO} \end{array}\right]⊖\left[\begin{array}{c} q_{CAM\_AUV}q_{j-1}^{AUV}{}^{-1}(p_{j}^{AUV}-p_{j-1}^{AUV})\\ {\left(q_{j-1}^{AUV}q_{CAM\_AUV}^{-1}\right)}^{-1}(q_{j}^{AUV}q_{CAM\_AUV}^{-1}) \end{array}\right] \end{array},$$

where $p_{j-1}^{VO},q_{j-1}^{VO}$ represent the position and attitude of the visual odometry at time j−1, and $p_{j}^{AUV},q_{j}^{AUV}$ represent the pose of AUV at time j. $q_{CAM\_AUV}$ is the quaternion corresponding to the transformation matrix from camera the coordinate to the AUV body coordinate. The symbol ⊖ is the subtraction of quaternions. The former term in the above formula is the pose increment of camera in adjacent two image frames in the visual odometry. The latter is to convert the pose increment of AUV into the corresponding camera pose increment. Thus, the incremental residual of camera’s pose between adjacent frames in camera coordinate at j−1 time is obtained. The first line represents the incremental position residual of cameras in adjacent frames, and the second line represents the incremental pose residual of cameras in adjacent frames.

The position $p_{j}^{D}$ of AUV relative to docking station is obtained according to USBL. The observed residuals generated by USBL are defined as:

(23) $$\begin{array}{ll} e_{j}^{USBL} & =z_{j}^{USBL}-f_{j}^{USBL}(X)\\ & =\;z_{j}^{USBL}-f_{j}^{USBL}(X_{j})\\ & =\;p_{j}^{D}-p_{j}^{AUV} \end{array},$$

The depth meter obtains the height $z_{j}^{Depth}$ of AUV relative to docking station, which is one-dimensional. The observation residuals generated by the depth meter are defined as:

(24) $$\begin{array}{ll} e_{j}^{Depth} & =z_{j}^{Depth}-f_{j}^{Depth}(X)\\ & =\;z_{j}^{Depth}-f_{j}^{Depth}(X_{j})\\ & =\;z_{j}^{Depth}-{\left[p_{j}\right]}_{3,1} \end{array},$$

where $z_{j}^{Depth}$ is one-dimensional, $p_{j}$ is three-dimensional, and ${\left[p_{j}\right]}_{3,1}$ is the value of its z-axis direction.

AHRS obtains the attitude $q_{j}^{AHRS}$ of AUV relative to docking station. The observed residuals generated by AHRS are defined as:

(25) $$\begin{array}{ll} e_{j}^{AHRS} & =q_{j}^{AHRS}-f_{j}^{AHRS}(X)\\ & =\;q_{j}^{AHRS}⊖q_{j}^{AUV} \end{array},$$

## 4. Results and Discussion of Experiments

**Figure 7:**
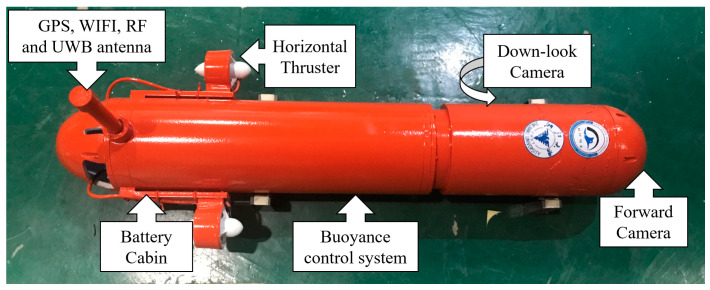
The AUV configuration in this work.

In order to verify the effectiveness of IU-ORBSLAM algorithm and multi-sensor information fusion localization algorithm proposed in this paper, the AUV is tested in the ZJU swimming pool. The AUV used is a torpedo type AUV with multiple degrees of freedom. The AUV is equipped with main circuit cabin, battery cabin, forward camera, down-look camera, horizontal thruster and buoyancy control system (Figure 7). Among them, the main circuit cabin is integrated with controller, circuit and various sensors. The depth meter is installed on the bottom of the AUV. The forward camera is used in optical navigation to track the guidance light. The down-look camera is used as the monocular visual odometer, though using an omnidirectional camera could greatly improve the robustness of pose estimation since it can ensure sufficient matching points [25]. Considering that the application scenario is underwater, the feature points are scattered on the bottom of the water. This is different from the situation of an autonomous vehicle on land, where the feature points are scattered on the two sides of the vehicle. The camera installed on bottom of the AUV only capture the feature points below it, so it is not necessary to use the omnidirectional camera. Both cameras have an opening angle of 170 degrees in this work. The AUV has four degrees of freedom which are complemented by two horizontal thrusters and the buoyancy control system. The GPS antenna, WIFI antenna, radio antenna and the antenna of UWB localization module are installed in the shell at the end of the AUV. The validation experiments were conducted at ZJU pool, which has a size of 50 m (length) × 21 m (width) × 1.5 m (depth). The water in the pool is fresh water which provides good visibility.

In order to verify the tracking effect of the proposed IU-ORBSLAM, AUV is tested in the swimming pool to collect data sets. The AUV travels with a velocity of 0.5 m/s and captures 15 images per second. While collecting the data set, the AUV is disturbed artificially to make AUV motion change. The datasets containing motion changes are used to test the ORBSLAM and IU-ORBSLAM algorithm. It is worth mentioning that the thresholds in tracking the last frame, tracking the key frame both and tracking the local map points are 50, 20 and 50, respectively. The number of tracking points after motion mutation is regarded as the evaluation standard of continuous working ability.

The number of feature points changes in ORBSLAM and IU-ORBSLAM when motion changes occur, as shown in Table 1. When motion mutation occurs in frame 0, the number of points tracking the last frame in ORBSLAM and IU-ORBSLAM are both below the threshold. According to the tracking logic in ORBSLAM, tracking the key frame is next executed. The number of the points tracking the key frame is bigger than the threshold, so the ORBSLAM will go on working. In the following frames, due to the influence of the mutation frame, the feature points of tracking the last frame is few and exist mismatching which results in pose estimated error and fewer correct matching points. Finally, in frame 7, the number of tracking the key frame is below the threshold which indicates tracking failure according to the tracking logic of ORBSLAM. While tracking based on the bags of words is executed at frame 0 and frame 1 where the number of the points tracking the last frame using motion model is below the threshold in IU-ORBSLAM. The RANSAC is utilized to eliminate mismatches next. So after mutation frame, the number of correct matching points of tracking the last frame using the motion model is almost bigger than that in ORBSLAM. With the movement, the number of feature points gradually increased and returned to normal. Results show that the tracking effect of IU-ORBSLAM is better than that of ORBSLAM when occurring motion mutation.

In order to verify the accuracy of the fusion localization algorithm, a fixed-trajectory navigation experiment was carried out in the swimming pool. The AUV travels a rectangle and a square trajectory three times, respectively. For the strong reflection of the pool, the USBL is simulated by GPS with similar measurement characteristics. The two sensors belong to the global localization sensor class that have low updating rates and some outliers. The localization error determined by the USBL and GPS sensor used in the test are both within 5 m. In order to make GPS work closer to USBL, GPS data was read every 3 s in the experiment. Therefore, it is reasonable to use GPS to simulate USBL localization. The localization information obtained by GPS is transferred into the AUV localization information in the docking coordinate according to Equation (9). The UWB module with high localization accuracy is used to record the accurate trajectory of the AUV which is regarded as the reference trajectory to be compared. The trajectory of USBL (GPS replaced) and localization based on multi-sensor information fusion is compared to the reference trajectory. The root mean square error (RMSE) of different trajectories and reference trajectories is calculated, and it is used to judge which method can obtain the trajectory closer to the reference trajectory.

**Figure 8:**
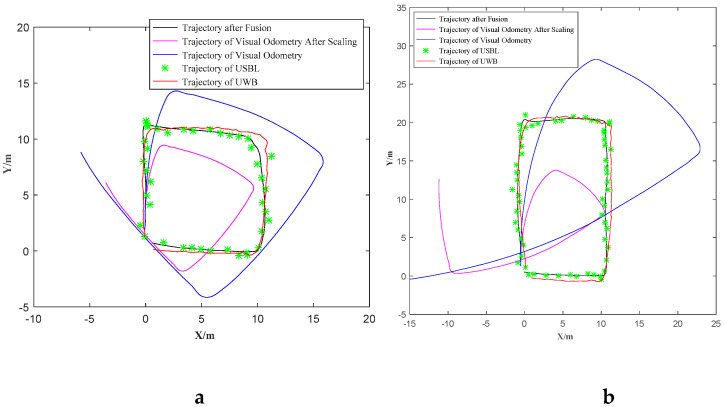
Fixed-trajectory navigation to test the localization performance. The AUV is control to travel along a square-shape (**a**) and triangle-shape (**b**) trajectory.

The AUV runs in the given trajectory which goes through the points (0 m, 0 m), (0 m, 10 m), (10 m, 10 m), (10 m, 0 m), (0 m, 0 m) in docking coordinates (as Figure 8a shows) three times. The localization performances are almost the same, so only one of the tests is discussed in this paper. The trajectories of visual odometry, USBL (GPS simulated), multi-sensor information fusion localization and UWB reference are shown in Figure 8. It can be found that in the visual odometry there exists an obvious angle accumulation error and it lacks displacement scale. The optimized scale coefficient is 0.610 after using multi-sensor information fusion localization. This scale coefficient means that the trajectory calculated by the visual odometer can be multiplied by this coefficient to get the trajectory closest to the actual AUV navigation. The root means square error (RMSE) of the USBL trajectory relative to UWB reference trajectory is 1.186 m, while the RMSE of multi-sensor information fusion localization trajectory relative to UWB reference trajectory is 0.299 m, which shows that the fusion trajectory is closer to the real trajectory. In addition, the localization information is updated every 0.1 s, so the proposed multi-sensor information fusion localization greatly improves the localization accuracy and reduces the influence of the outliers.

In addition, the AUV runs in the given trajectory which go through the points (0 m, 0 m), (0 m, 20 m), (10 m, 20 m), (10 m, 20 m), (10 m, 0 m), (0 m, 0 m) in docking coordinates (Figure 8b) three times. One of the tests is shown in the paper. The optimized scale coefficient is 0.465. Taking the UWB reference trajectory as the real trajectory, the RMSE of the USBL trajectory relative to the UWB reference trajectory is 1.455 m, and the RMSE of multi-sensor information fusion localization trajectory relative to the UWB reference trajectory is 0.395 m. It is still proved that the localization accuracy after the proposed multi-sensor information fusion is greatly improved.

**Figure 9:**
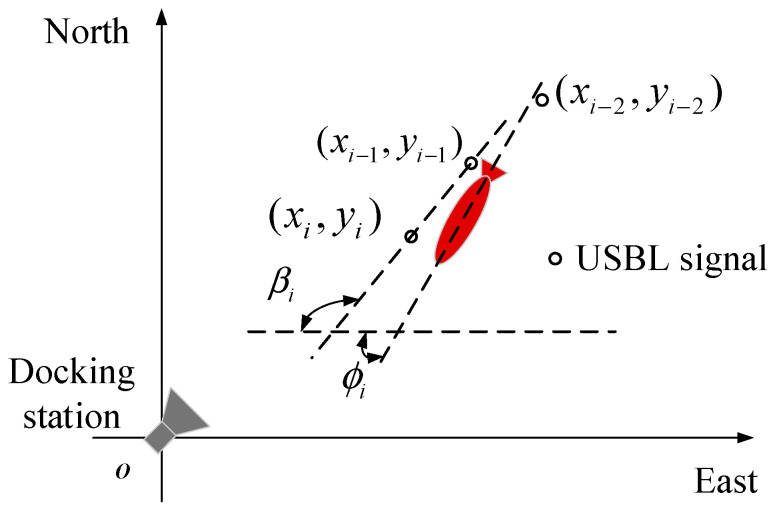
Schematic diagram of AUV docking localization initialization.

The purpose of this paper is to fuse USBL, monocular odometer, AHRS and depth meter data to improve the performance of the AUV in the transition between homing and docking. Five docking missions were carried out in the swimming pool. The AUV starts from about 30 m away from the docking station and enters the transition between homing and docking. The AUV enters the transition from the place with a large lateral deviation of 5 m which means the AUV has more difficulty in completing these docking missions. The forward velocity of the AUV is 0.5 m/s and the localization updating rate is 10 Hz. Five consecutive docking missions were carried out. If the consecutive five docking missions can be all successfully completed, we believe that the success of the proposed method. When the AUV goes into 10 m in front of the docking station, the forward camera can see the guidance light, and the optical navigation using one camera and one light is applied to guide the AUV entering the docking station. In the transition, due to the existence of USBL outliers, the initial position of AUV after entering the transition is not accurate enough. At this time, only relying on the fusion algorithm could not correct the localization to the ideal accuracy range in a timely way. Therefore, in the transition, it is necessary to have a more accurate USBL localization signal to initialize the position, so as to avoid using abnormal USBL localization data to cause the AUV to deviate from the correct track. Therefore, an initialization method is used (Figure 9) [22]. The discriminant is:

(26) $$|\pi +\phi_{i}-\arctan(\frac{y_{i}-y_{i-1}}{x_{i}-x_{i-1}})|\leq \varepsilon ,$$

where, ε is the threshold, which equals to π6 in the paper. $\phi_{i}$ is the heading obtained from the AHRS. When the USBL data of two consecutive points can meet Equation (26), the latest USBL localization data is considered to meet the initialization requirements. In order to initialize according to the above method, it is necessary to make the AUV navigate according to the fixed heading angle until the feasible initial localization is obtained, and then track the relevant mission points. The trajectories in Figure 9 are all obtained after this initialization.

**Figure 10:**
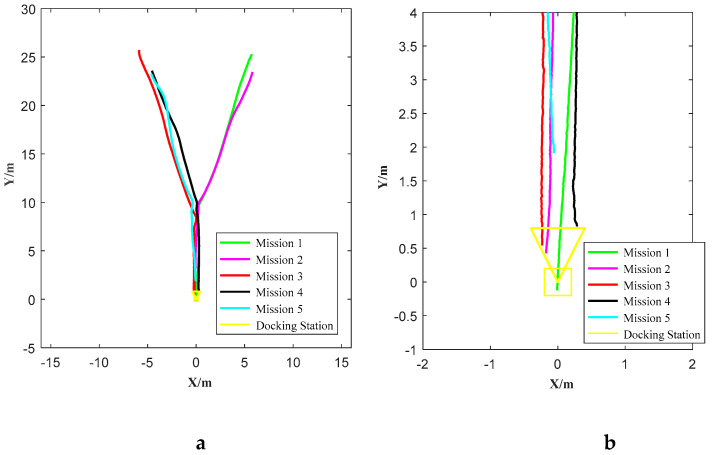
Trajectories of five docking missions. The image b is the trajectory enlarged in terminal docking.

**Figure 11:**
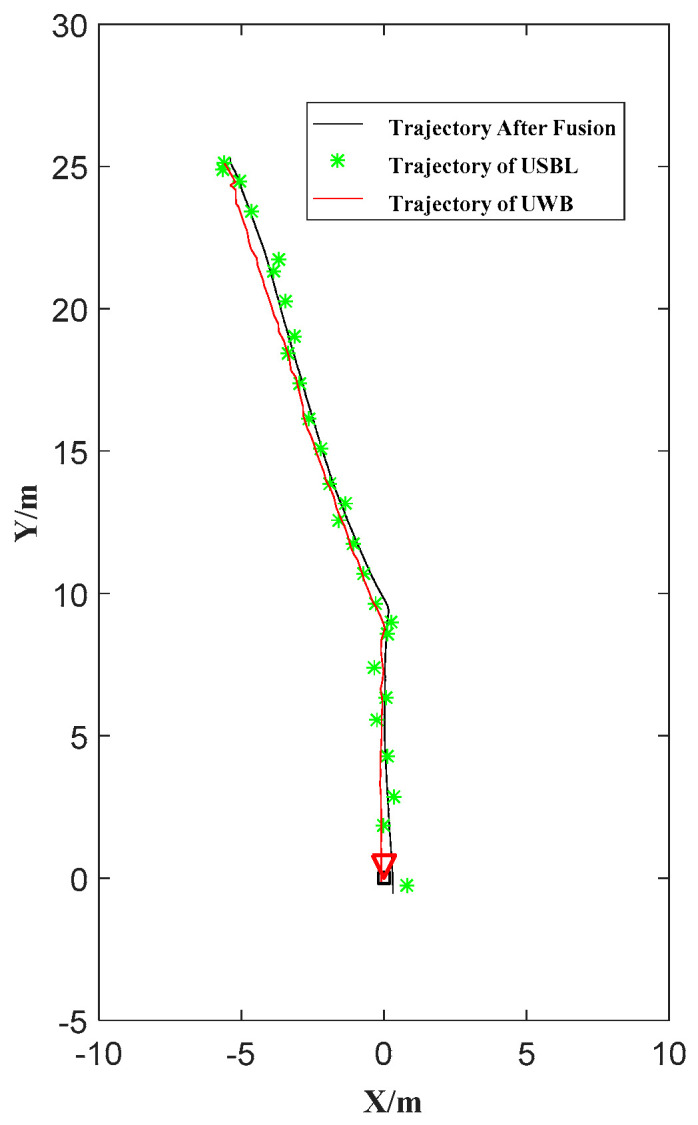
Trajectories comparison in one navigation docking.

**Figure 12:**
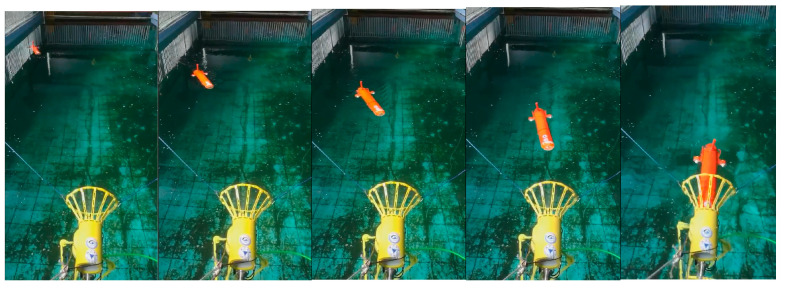
AUV docking images sequence.

The trajectories of five docking missions are shown in Figure 10. The enlargement of the terminal trajectory is shown in Figure 10b. All five consecutive docking missions go into the entrance of the docking station, that is, the rate of successful docking is 100%. The largest lateral deviation is 0.31 m, which does not exceed half the width of the docking station (0.4 m). One of the successful docking trajectories is shown in Figure 11. The corresponding images sequence of AUV docking is shown in Figure 12. There is value jitter in trajectory of USBL, while the trajectory after information fusion is relatively smooth and is almost consistent with the reference trajectory. The result proves that localization performance of the localization method based on multi-sensor information fusion is improved, and the visual odometry can smooth out the impact of USBL localization value jitter.

**Figure 13:**
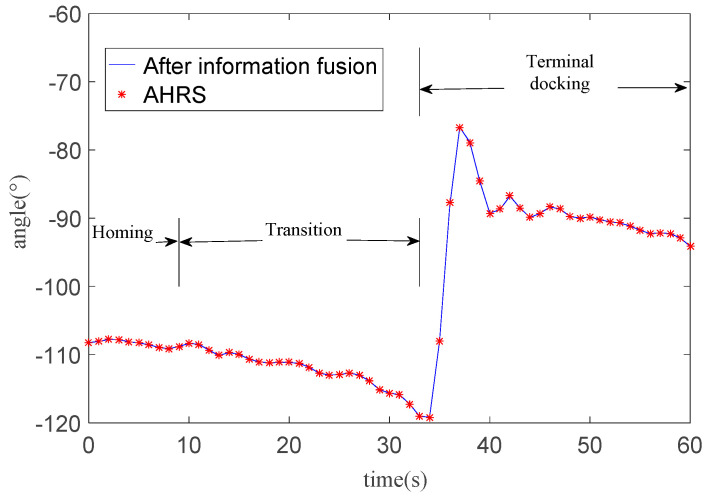
AUV heading changes in one docking mission.

In addition to the change of AUV position information, another parameter we focus on is the change of AUV heading angle. As Figure 13 shown, the AUV goes into the transition at 9.5 s. At 33 s, the AUV distinguishes the guidance light and starts the optical navigation. The AUV adjusts its heading from −120 degrees to −90 degrees. The overshoot heading angle is about 15 degrees which means that there is a dramatic change in heading when the AUV starts the optical navigation.

The AUV heading tends to stabilize gradually after 10 s of oscillation. The RMSE of heading angle relative to AHRS heading angle is 0.067 degrees. Combined with Figure 11, the obvious adjustment occurs at that 10 m in front of the docking station during. Even though the AUV heading changes greatly, the angle after fusion is still very close to that measured by AHRS.

## 5. Conclusions

The docking technology of AUVs is one of the most important technologies in underwater observation. In view of the problems existing in docking navigation, an IU-ORBSLAM is proposed based on ORBSLAM. IU-ORBSLAM uses bags of words and RANSAC to improve tracking performance of visual odometry in adjacent frames when motion changes and light variation occurs. On this basis, a redundant multi-layer tracking framework is constructed. The continuous tracking ability of IU-ORBSLAM in an underwater environment is proved by swimming pool tests. Since the monocular odometer in IU-ORBSLAM can only obtain scale-free relative pose changes from two adjacent frames, there is a cumulative error (or cumulative drift). Therefore, it is not enough to only use monocular vision in AUV docking, but one also needs to fuse with information of other sensors. This paper presents a multi-sensor information fusion localization method based on nonlinear optimization for underwater monocular visual odometry, USBL, depth meters and AHRS. The sum of the residual of each sensor is taken as the cost function to optimize the AUV’s pose and scale coefficient of monocular visual odometry. USBL is used to provide scale information for monocular visual odometry and to suppress the cumulative drift of monocular visual odometry. Monocular visual odometry is applied to improve localization updating rate and reduce the influence of outliers measured by USBL. According to the swimming pool fixed-trajectory navigation tests, the localization accuracy after fusion is higher than that before fusion. In order to verify the effectiveness of the proposed localization algorithm in AUV docking, five docking missions were conducted in a swimming pool. The results show that AUV has successfully entered the docking station in each docking mission, which verifies the feasibility of the localization method based on multi-sensor information fusion. The main contributions of this paper can be summarized as follows:

(1) To improve the ability of the ORBSLAM working underwater, an improved underwater ORBSLAM (IU-ORBSLAM) in the transition between AUV homing and docking is proposed, which has better continuous working ability in the situation of light and motion changes than ORBSLAM.
(2) A nonlinear optimization based multi-sensor information fusion localization method is proposed, which includes the information of monocular visual odometry in IU-ORBSLAM, USBL, AHRS and depth meters. It solves the scale problem of the monocular odometer and improves the localization accuracy and update rate in the transition, which can increase the docking performance.

In future work, this localization method will be applied to a wider area of water for lake experiments. Because visual odometry is used in this method, it is necessary to study the influence of water turbidity on the performance his localization method.

## Figures and Tables

**Table 1:** The number of tracking points changes when occurring motion mutation in ORBSLAM and IU-ORBSLAM.

	ORBSLAM			IU-ORBSLAM			
Frame ID	Track the Last Frame by Motion Model	Track the Key Frame	Track Local Map Points	Track the Last Frame by Motion Model	Track the Last Frame by Bags of Words	Track the Key Frame	Track Local Map Points
−2	196	-	352	202	-	-	350
−1	188	-	355	188	-	-	361
0	13	27	84	12	59	-	79
1	0	69	162	0	60	-	119
2	69	-	121	50	-	-	116
3	55	-	115	51	-	-	281
4	48	29	86	140	-	-	240
5	41	22	77	137	-	-	210
6	47	25	56	111	-	-	173
7	26	13 (failure)	-	104	-	-	263

## Data Availability

Not applicable.
